# Cost Effectiveness of Different Treatment Strategies in the Treatment of Patients with Moderate to Severe Rheumatoid Arthritis in China

**DOI:** 10.1371/journal.pone.0047373

**Published:** 2012-10-09

**Authors:** Bin Wu, Alisa Wilson, Fang-fang Wang, Su-li Wang, Daniel J. Wallace, Michael H. Weisman, Liang-jing Lu

**Affiliations:** 1 Clinical Outcomes and Economics Group, Department of Pharmacy, Renji Hospital, Shanghai Jiaotong University School of Medicine, Shanghai, People's Republic of China; 2 Division of Rheumatology, Cedars-Sinai Medical Center, Los Angeles, California, United States of America; 3 Department of Rheumatology, Renji Hospital, Shanghai Jiaotong University School of Medicine, Shanghai, People's Republic of China; Groningen Research Institute of Pharmacy, United States of America

## Abstract

**Background:**

To analyse the cost-effectiveness of traditional disease-modifying anti-rheumatic drugs (tDMARDs) compared to biological therapies from the perspective of Chinese society.

**Methodology/Principal Findings:**

A mathematical model was developed by incorporating the clinical trial data and Chinese unit costs and treatment sequences from a lifetime perspective. Hypothetical cohorts with moderate to severe RA were simulated. The primary outcome measure–quality-adjusted life years (QALYs)–was derived from disease severity (HAQ scores). Primary analysis included drug costs, monitoring costs, and other costs. Probabilistic and one-way sensitivity analyses were performed. Treatment sequences that included TNF antagonists and rituximab produced a greater number of QALYs than tDMARDs alone or TNF antagonists plus DMARDs. In comparison with tDMARDs, the incremental cost-effectiveness ratios (ICERs) for etanercept, infliximab, and adalimumab without rituximab were $77,357.7, $26,562.4 and $57,838.4 per QALY and $66,422.9, $28,780.6 and $50,937.6 per QALY, for etanercept, infliximab, and adalimumab with rituximab. No biotherapy was cost-effective under the willingness to pay threshold when the threshold was 3 times the per capita GDP of China. When 3 times the per capita GDP of Shanghai used as the threshold, infliximab and rituximab could yield nearly 90% cost-effective simulations in probabilistic sensitivity analysis.

**Conclusions/Significance:**

tDMARD was the most cost-effective option in the Chinese healthcare setting. In some relatively developed regions in China, infliximab and rituximab may be a favorable cost-effective alternative for moderate to severe RA.

## Introduction

Rheumatoid arthritis (RA), with a prevalence rate unmet of 0.2% to 0.37% in China [1], [2], is a systemic autoimmune disease that causes chronic inflammation of the joints and tendons resulting in progressive bony erosions and joint damage. Disability and premature mortality caused by RA have substantial socioeconomic implications [3]. Disease modifying antirheumatic drugs (DMARDs), such as methotrexate (MTX), may relieve symptoms and delay disease progression. As a result, DMARDs are often recommended as first-line therapy for RA either in succession or combined with other anti-inflammatory agents [4]. However, when treatment efficacy with these regimens declines, patients usually need to switch regimens or the disease becomes more active and progressive. Licensed biological agents, such as tumor necrosis factor (TNF)-α inhibitors, the costimulatory molecule inhibitor (abatacept), the B-cell depletion agent (rituximab), and the interleukin-6 receptor inhibitor (tocilizumab), have greatly enhanced effective RA treatment and improved health outcomes [4], [5], [6].

Etanercept, infliximab, and adalimumab, which have been used in Chinese RA patients, are biological agents that bind and block TNF. Etanercept is a soluble TNF receptor fusion protein that interferes with both TNF-α and TNF-β, while adalimumab and infliximab are monoclonal antibodies against TNF-α [7]. The results of clinical trials demonstrate that all TNF blockers are able to slow the progression of joint damage and alleviate clinical symptoms in many patients with RA, especially when used in combining with traditional DMARDs (tDMARDs). Although the health benefits achieved by the TNF inhibitors are notable, the high price of these agents preclude their widespread prescription in China. Traditional DMARDs, non-steroidal anti-inflammatory drugs (NASIDs), and corticosteroids still play a primary role in Chinese clinical practice for the treatment of RA, even moderate to severe RA. At present, patients who have an inadequate response to tDMARDs, must pay out-of-pocket costs for biological therapy. To fill the unmet efficacy of tDMARDs in China, biological agents likely need to be covered by the healthcare system.[8] However, the higher costs of biological agents in comparison with traditional treatments would considerably increase the resource budget for RA treatment. To use biological therapy or tDMARDs is an urgent question for RA patients in this resource-limited setting. Dozens of studies on the cost-effectiveness of biological treatments for RA have been reported [3]. However, these studies almost came from developed countries, and few RA-focused economic evaluations to guide treatment decisions in health resource-limited setting.

Reasons for the rarity of comparative health economic data for RA in health resource-limited settings include the absence of funds for the implementation of clinical trials with large cohorts over the long term. Due to their lower cost, modeling techniques are widely used to estimate the relative health and economic outcomes of competing treatment strategies. Mathematical models simulate the disease course by incorporating published clinical data and measuring the input and output based on the cost and effectiveness in a given region. At the same time, computer simulation technology allows the modeling approach to compare competing strategies with a ‘virtual’ head-to-head modality. Economic analyses based on mathematical models have been widely used to evaluate RA treatment worldwide [9]. This study aims to evaluate the cost–utility of different treatment strategies after treatment failure with at least two tDMARDs in a Chinese setting, a health resource-limited region.

## Patients and Methods

### Model Overview

This analysis uses a Markov cohort model programmed in R software environment (version 2.13.1; R Development Core Team, Vienna, Austria),in which the lifetime costs and health benefits of the introduction of different treatment strategies were measured for identical and hypothetical RA patient cohorts, which were assumed to have refractory response to at least two tDMARDs, one of which was methotrexate. The baseline characteristics of the hypothetical RA cohorts are based on the published studies, which had an mean age of 49 years, mean weight of 65 kg, 85.6% of female and the mean health assessment questionnaire (HAQ) score of 1.6. [10], [11]. The cycle length of the model is 6 months; this cycle is consistent with recommendations for DMARD assessment [4]. At the completion of each cycle, patients may move to the next therapy in the treatment sequence or to death.

**Figure 1:**
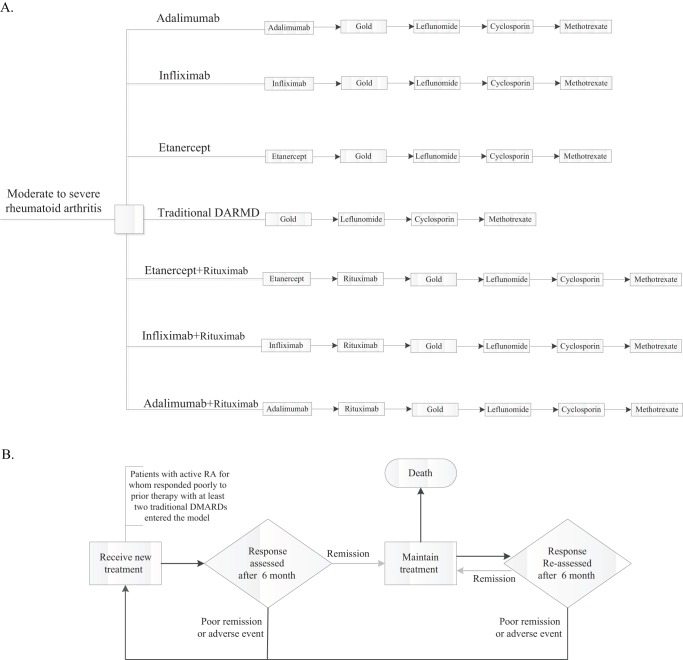
Model architecture: (A) model treatment sequences, and (B) decision analysis for the treatment.

The clinical treatment pathway of the model follows current clinical practice in China. At present, four biological agents (etanercept, infliximab, adalimumab, and rituximab) have been approved for treating RA in China. According to the opinion of Chinese rheumatologists, the sequential use of rituximab is currently permitted when the patient has an inadequate response to TNF-α inhibitors. Consequently, it is essential to analyze the economic outcomes of switching to rituximab after the failure of TNF inhibitors. The hypothetical patient would receive one of the following seven competing strategies to manage active moderate to severe RA: tDMARDs only (tDMARD strategy), initiation with etanercept followed by tDMARD (etanercept strategy), initiation with infliximab followed by tDMARD (infliximab strategy), initiation with adalimumab followed by tDMARD (adalimumab strategy), etanercept therapy followed by rituximab and tDMARD (etanercept + rituximab strategy), infliximab therapy followed by rituximab and tDMARD (infliximab + rituximab strategy) and adalimumab therapy followed by rituximab and tDMARD (adalimumab + rituximab strategy). The sequence of these seven strategies is shown in Figure 1A. During the course of biotherapy, concomitant use of methotrexate was recommended [4]. Once patients enter the model and receive one cycle treatment, the treatment response is accessed and non-responders are permitted to withdraw from the current treatment and switch to a new treatment in the treatment sequence. Patients achieving American College of Rheumatology criteria response (ACR20/ACR50/ACR70) remain on the current treatment until the efficacy diminishes or intolerable adverse events occur (Figure 1B). The model assumes that an ACR response leads to an improvement in HAQ scores and that HAQ score in non-responders deteriorate and the disease relapses. If all treatments in the sequence fail, we assumed that palliative therapy was initiated.

The perspective on costs and outcomes adopted in this analysis is the Chinese healthcare system. Direct medical costs in the economic evaluation were estimated. HAQ scores are converted to utility values. Drug and other healthcare costs are included. Average quality-adjusted life years (QALYs) and costs for each strategy were measured and the primary endpoint was the incremental cost per QALY for biological strategy in comparison with tDMARD strategy. Costs and outcomes were discounted by an annual rate of 3%.

### Clinical Data

#### Efficacy

The primary RA treatment goal aims toward controlling the inflammatory activity of the disease, preventing the progression of joint damage, enhancing physical function and quality of life and, if possible, disease remission [4]. Although response criteria and clinical remission may be assessed using the 28 joint count Disease Activity Score (DAS28), few clinical trials use the DAS28 as the primary endpoint. All clinical trials, though, have used the ACR response criteria. Unfortunately, the ACR response criteria are only a relative index and thus not a perfect endpoint for decision making. However, in order to estimate utility values for the different strategies used in the model, ACR response rates were used as the primary endpoint. Published reports have suggested that results between the ACR and DAS were similar [10].

ACR response evidence was obtained from a review of published reports in patients with moderate to severe RA. Since no clinical trial directly provided the comparative results of all strategies, an indirect comparison of all treatments was conducted by a well-established method [10]. Because the control arm in all published trials received MTX, the variations of response rates in different trials could be adjusted by using reference placebo response rates. The following is the formula for calculating adjusted response: RP+(1-RP)×([TT-TP/[1-TP]), RP, TT and TP was indicated for the response of reference placebo, trial treatment, and trial placebo, respectively [10]. To minimize bias, placebo response rates from two adalimumab randomized controlled trials (RCT) were extracted for calculating the weighted mean response rate as the reference placebo response rate, because one of the two adalimumab RCTs for moderate to severe RA was larger and more comprehensive than similar designed etanercept and infliximab trials [12], [13], [14], [15]. The ACR response data was derived from the Study for Understanding Rituximab Safety and Efficacy (SUNRISE)trial, which was the first RCT phase III trial assessing fixed interval retreatment of rituximab. This trial provides greater clarity on the use of subsequent courses of rituximab among patients with moderate to severe active RA with previous inadequate response to TNF inhibitors [16]. ACR response rates for tDMARDs as third and fourth line use are limited. The response data used in this analysis are derived from other published pharmacoeconomic reports [17]. The unadjusted and adjusted ACR response data are shown in Table 1.

**Table 1:** ACR response and withdrawal probability of treatments.

Treatment	ACR20/50/70 response (%)			Withdraw&		Source
	Unadjusted value	Adjusted value	Distribution	Value	Distribution	
Reference of placebo	RP: (29.5/9.5/2.5) and (14.5/8.1/4.8)	RP: (25.7/9.1/3.1)#	Dirichlet			[14], [15]
Etanercept	TT: (71/39/15) TP: (27/3/0)	TT: (70.5/42.9/17.6) *	Dirichlet	scale = 0.15 shape = 0.6276	Weibull	[12], [18]
Infliximab	TT: (50/27/8) TP: (19/5/0)	TT: (54.2/30.2/10.8) *	Dirichlet	scale = 0.2758 shape = 0.5775	Weibull	[13], [18]
Adalimumab	TT: (63.3/39.1/20.8) and (67.2/55.2/26.9)	TT: (64.2/42.8/22.2) #	Dirichlet	scale = 0.2107 shape = 0.5531	Weibull	[14], [15], [18]
Rituxiumab	(48/27/11)	NA	Dirichlet	First cycle:52% subsequent cycle:9%	Beta	[16]
Leflunomide	(37/0/0)	NA	Dirichlet	20.7%	Beta	[17]
Gold	(37/0/0)	NA	Dirichlet	10.6%	Beta	[17]
Cyclosporin	(48/0/0)	NA	Dirichlet	25.3%	Beta	[17]

NA: Not applicable.

# It was calculated by weighted average methods: (Response_1_ × n_1_ + Response_2_ × n_2_)/(n_1_+n_2_). Response_1_ and Response_2_ were indicated as the response in control arm of trial 1 and 2, and n_1_ and n_2_ were the patient number, respectively. [14], [15].

* Adjusted response  = RP + (1-RP) × ([TT-TP]/[1-TP]), RP, TT and TP was indicated for the response of reference placebo, trial treatment, and trial placebo, respectively. [10].

& Withdraw probability (t)  = 1 – exp(λ× (t–1) ^γ^–λ×t^γ^). In this formula, t was indicated as the current cycle number, and λ and γ were scale and shape parameters, respectively.

#### Long term withdrawal

The long term probability of withdrawal for etanercept, infliximab, and adalimumab was taken from the eight years of surveillance of clinical practice in the nationwide Danish DANBIO registry [18]. To extrapolate the probability of withdrawal beyond the follow-up period, two-parametric Weibull survival models were used to fit the data extracted from the drug survival rate curves [19]. This method has been widely used in fitting survival data. The shape parameter (γ) allows the hazard function to increase or decrease with increasing time; if γ>1.0, the hazard rate strictly increases in a nonlinear pattern with increasing time. The scale parameter (λ) is related to the measurement unit of time. The estimated scale and shape parameters are presented in Table 1. The probability of withdrawal for other treatment was derived from published reports [16], [17].

#### Mortality

Natural mortality could be incurred by RA patients at any point on the treatment pathway. The model used a normal life table from the life tables for WHO member states (2011) to adjust mortality risk for patients with RA(1.33 per unit HAQ) [20], [21].

### Quality of life effect

Utility scores are essential for adjusting life expectancy with quality of life and generating QALYs for performing cost-utility analysis. In the current analysis, we assumed HAQ scores had a credible bridge with utility values. Utility values were estimated by patients' HAQ scores using the following linear transformation formula: utility values (HUI-3) = 0.76–0.28×HAQ+0.05×FEMALE [22]. The improved HAQ score achieved by treatment was converted based on ACR response status: −0.1, −0.45, −0.85 and −1.11 for non-responders, ACR20, ACR 50, andACR70, respectively [23]. During treatment and palliative care, we assumed a constant progression of disability over time and HAQ-score increases of 0.017and 0.065 per each 6-month cycle for treatment and palliative care, respectively [17], [24].

### Resource use and costs

All costs are reported in 2011 US dollars ($). The following direct medical cost components were taken into account: drug, monitoring and administration, toxicity, and inpatient care. The costs were estimated from Chinese healthcare systems.

The prices of medical services in China are far lower than in Western countries. For example, general nursing services for inpatient care does not exceed $3 per day in China, which is lower than most countries [25]. Drug costs were the largest contributor to overall healthcare costs (Table 2). To estimate drug costs, the treatment schedules were assumed to follow labeled standard protocols: the treatment schedule for etanercept is 50 mg i.h. per week; for infliximab the treatment schedule is 3 mg/kg i.v. on weeks 0, 2, 6 and then every 8 weeks; for adalimumab the treatment schedule is 40 mg s.c. every other week, and for rituximab the treatment schedule is 1000 mg i.v. on weeks 1 and 2 weeks every 9 months with premedication with methylprednisolone 100 mg i.v. before each infusion [16]. Costs for subcutaneous or intravenous administration are estimated from local hospitals, which are made up of the cost of medical service, medical consumable materials associated with administration and intravenous solutions, etc For tDMARD, including gold, leflunomide, cyclosporine and methotrexate, p.o. was the primary method of administration, so administration costs were omitted from the analysis.

**Table 2:** Estimated costs and resource use.

Resource	Cost per quantity($)	Rang	Utilization	Distribution
Etanercept	378.46 per 25 mg	340.62∼416.31*	50 mg i.h. or i.v. per week	fixed
Infliximab	1015.38 per 100 mg	913.85∼1116.92*	3 mg/kg i.v. on weeks 0, 2, 6 and then every 8 weeks(three vials required for a 65 kg patient)	fixed
Adalimumab	1215.38 per 40 mg	1093.85∼1336.92*	40mg subcutaneous injection every other week	fixed
Rituxiumab	605.77 per 100 mg	545.19∼666.35*	1000 mg on week 1 and 2	fixed
Leflunomide	0.74 per 10 mg	0.54∼1.23*	10 mg per day for Chinese patient,orally	lognormal
Gold	0.57 per 3 mg	0.52∼0.63	3 mg twice a day, orally	fixed
Cyclosporin	0.91 per 25 mg	0.77∼1.23	3 mg/kg per day for a 65 kg patient,orally	lognormal
Methotrexate	0.03 per 2.5 mg	0.02∼0.05	12.5 mg per week, orally	lognormal
Methylprednisolone	4.32 per 40 mg	3.08∼4.62	100 mg i.v. before infliximab each infusion	lognormal
Outpatient visit	98.22 per cycle	46.24∼123.11#		lognormal
Inpatient	184.62 per patient per day	92.31∼230.77#	For patinets with 0.0<HAQ score <0.5, inpatient day was 0.68, 0.6<HAQ score <1.0 was 2.77, 1.1<HAQ score <1.5 was 4.12, 1.6<HAQ score <2.0 was 8.86, 2.1<HAQ score <2.6 was 10.25 and HAQ score <3.0 was 4.56	lognormal
Administration for i.v.	7.69 per time	4.62∼15.38#		lognormal
Average wage	21.9	6.11∼26.79$		normal

* The range was assumed for one-way sensitivity analysis.

# The range was estimated from local hospitals.

$ The range was derived from Chinese National Bureau of Statistics.

Outpatient visits (OPV) are essential for RA patients. However, adherence to a regular schedule of OPVs in China is unsatisfactory due to the fact that health resources for examinations and laboratory tests are limited. To estimate the cost of OPVs for Chinese RA patients, we measured the average cost of an OPV per patient per 6-months in local hospitals after excluding the cost of medications based on expert opinions. The reason excluding the cost of medication for RA was that the cost of medication had been dependently measured in each cycle by the methods descripting in the last paragraph. The cost of OPVs included the examination with X-ray, routine blood test, biochemical laboratory test, and medical services. The cost for OPVs per patient per cycle is shown in Table 2.

The cost for inpatient treatment is estimated on the basis of stratified HAQ score, which was strongly correlated with deterioration of physical function and increase in health resource expenditure [26], [27]. Because of the paucity of published evidence of this relationship for China, the relationship between HAQ scores and inpatient days were assumed to be similar with the Swedish 5-year observational study of 116 consecutive RA patients [26]. The average cost of an inpatient day was estimated by local rheumatologists.

Productivity losses due to RA are estimated in the analysis by the approach of inpatient cost evaluation. This method measures productivity loss by stratifying HAQ-score groups. Based on the data derived from the Chinese National Bureau of Statistics, the daily productivity losses in year 2010 were, on average, $21.9 in China.

### Sensitivity analyses

Uncertainty around the model's key parameters was assessed by employing one-way sensitivity and probabilistic sensitivity analyses. In one-way sensitivity analyses, upper and lower boundaries for parameters were changed to evaluate the impact of parameters on the robustness of the model. The upper and lower boundaries of proportions and probabilities were assumed as 90%×base case and 110%base case, respectively. Ranges of costs were showed in Table 2. Ranges of mean age, initial HAQ score and discount rate were from 30to 60 years, 1.1 to 2.5 and 0.1% to 8%, respectively. Distributions described in Table 1 and 2 were adopted to the corresponding parameters and values sampled by 1000 Monte Carlo simulation for jointly examining the uncertainty in all model parameters with an assumed standard deviation of 10% from mean values. We used 3×the per capita GDP of China ($11,034)/QALY and 3×the per capita GDP of Shanghai City ($38,376)/QALY as the threshold according to WHO recommendation [28], [29]. We varied endpoint (ACR20, 50 and 70) of treatment for scenario analysis and repeated the probabilistic analyses to assess their effect on the ICER.

## Results

### Base-case Analyses

**Figure 2:**
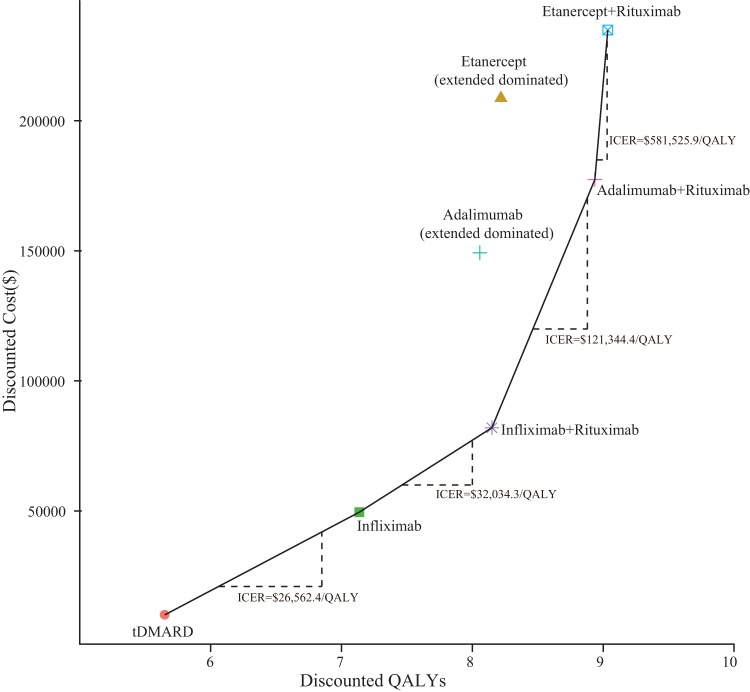
The cost-effectiveness frontier indicates the most efficient options among the seven competing treatment strategies.

Table 3 and Figure 2 report the results of the base-case analyses for each strategy. When only tDMARDs are prescribed for RA patients, the estimated lifetime mean costs and QALYs are $10,037.1 and 5.65. Induction of TNF-inhibitors produced greater QALYs and expended more health resources in comparison with the tDMARD strategy. The etanercept strategy gained the greatest marginal QALYs(2.57) and highest marginal cost ($198,696.7), followed by the adalimumab strategy(2.41QALYs and $139,240.5)and infliximab(1.49QALYs and $39,482.8). After the failure of TNF-inhibitors, adding rituximab to etanercept increases the health benefit and cost by0.82marginalQALYsand $26,161.7in comparison with the etanercept strategy alone. The addition of rituximab to infliximab increases the marginal QALYs and cost by 1.01and $32,462.3 compared to infliximab alone. Finally, adding rituximab to adalimumab results in a marginal QALY increase of 0.88 and a marginal cost increase of $28,163.8compared to adalimumab alone.

**Table 3:** Cost-effectiveness results of the base-case analyses.

Strategies	Drug cost ($)	Direct cost ($)	Total cost ($)	Total QALYs	Incremental cost	Incremental QAYLs	ICER with productivity lose*	ICER without productivity lose*
tDMARD	4,064.8	9,191.8	10,037.1	5.65	–			
Etanercept	198,944.0	207,981.7	208,733.8	8.22	198,696.7	2.57	77,357.7	77,394.0
Infliximab	44,998.6	49,048.2	49,519.9	7.14	39,482.8	1.49	26,562.4	26,813.8
Adalimumab	143,228.7	148,642.2	149,277.6	8.06	139,240.5	2.41	57,838.4	57,925.6
Etanercept+ Rituxiumab	224,671.3	234,143.5	234,895.5	9.04	224,858.4	3.39	66,422.9	66,450.4
Infliximab+ Rituxiumab	76,925.2	81,510.5	81,982.2	8.15	71,945.1	2.50	28,780.6	28,930.1
Adalimumab+ Rituxiumab	170,926.3	176,806.0	177,441.4	8.94	167,404.3	3.29	50,937.6	51,001.5

* tDMARD strategy was the baseline comparator.

The cost-effectiveness frontier indicates the most efficient strategies and their respective ICERs among the 7 competing treatment alternatives (Figure 2). The most efficient strategy is infliximab with an ICER of $26,562.4 compared with tDMARD, followed by infliximab + rituximab, adalimumab + rituximab, and etanercept + rituximab with an ICER of $32034.3, $121,344.4 and $581,525.9 compared with infliximab, infliximab + rituximab and adalimumab + rituximab, respectively. The etanercept and adalimumab strategies are extended dominated.

Drug costs for the tDMARD strategy were nearly 40.5%of the total cost, and the proportions for the remaining 6 biotherapies were all over 90%. This was expected given that the costs of medical service, laboratory tests, and productivity are far lower than the cost of drugs. The exclusion of productivity losses resulted in lower cost estimates for tDMARD and the other 6 biotherapy strategies (from $9,191.8in tDMARD strategy to $234,143.5 in etanercept + rituximab strategy) with higher ICER(from $26,813.8/QALY in infliximab strategy to $77,394.0/QALY in etanercept strategy) compared to tDMARD strategy (Table 3).

### Sensitivity Analyses

**Figure 3:**
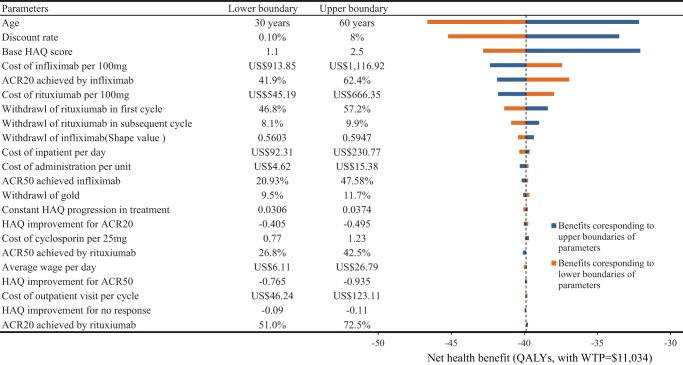
Tornado diagram representing the net health benefit (QALYs, with WTP = $11,034) in univariate sensitivity analysis for infliximab + rituximabvs tDMARD strategy in changing baseline parameters. The width of the bars represents the range of the results when the variables were changed. The vertical dotted line represents the base-case results.HAQ  = Health Assessment Questionnaire; ACR  = American College of Rheumatology criteria.

The robustness of the results was examined by conducting one-way sensitivity analyses. The results are shown in Figure 3. In examining the effect that age at treatment has on the net health benefit of the infliximab + rituximab strategy, older age leads to more health benefits. Adjusting the age from 30 to 60 years resulted in the greatest effect, changing the net health benefits from −46.6QALYs to −32.1QALYs. Because the drug cost is the a major contributor to the total cost, it was expected that the result was second most sensitive to changing the discount rate from 0.1% to 8%: increasing the discount rate to 8%resulted in an additional 33.5QALYs in comparison with the base-case results; while decreasing the discount rate to 0.1%, an additional −45.2 QALYs was gained in comparison with the base-case results. Base HAQ score at the time of biotherapy initiation was different; the result of one-way sensitivity analyses showed the net health benefit increased with the increased base HAQ score from 1.1 to 2.5, which was the third most influential variable. Efficacy of infliximab was varied in different cohorts; the result of one-way sensitivity analyses showed that varying the ACR20 of infliximab from 46.8% to 57.2% was the fourth most influential variable [30], [31]. Changing the cost of infliximab and rituximab by ±10%caused changes in the net health benefits for infliximab + rituximab strategy that ranged from −6.2% to 6.2% and −4.8% to 4.8% of the base case result, respectively (figure 3). Other import effect factors included the withdrawal of infliximab and rituximab and cost of inpatient per day, *et* *al.* Other parameters, such as the cost and efficacy of tDMARDs, have a relatively small impact on the net health benefit of infliximab + rituximab strategy over tDMARDs strategy.

**Figure 4:**
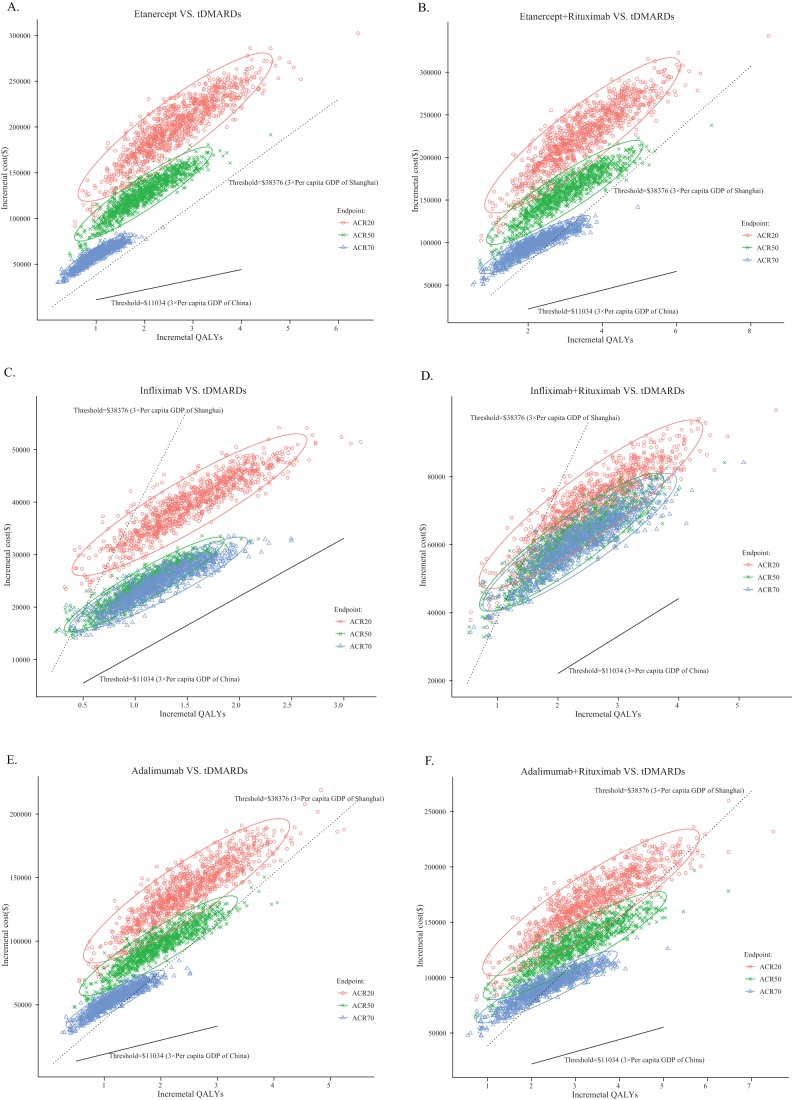
Probabilistic scatterplot of the incremental cost-effectiveness ratio (ICER) between tDMARD and (A) Etanercept, (B) Etanercept+Rituxiumab, (C) Infliximab, (D) Infliximab+Rituxiumab, (E) Adalimumab and (F) Adalimumab+Rituxiumab for a cohort of 1,000 moderate to severe RA patients withan ACR20, ACR50, and ACR70 endpoint, respectively. The x-axis and y-axisrepresent lifetime incremental QALYsand costs, respectively. Each dot represents the ICER for 1 simulation. Ellipse surrounds 95% of estimates. The solid and dashed lines represent the cost-effectiveness threshold of 3×the per capita GDP of China and Shanghai per QALY gained, respectively. Dots that located below the ICER threshold representcost-effective simulations for the active strategy compared with the tDMARD strategy.

**Figure 5:**
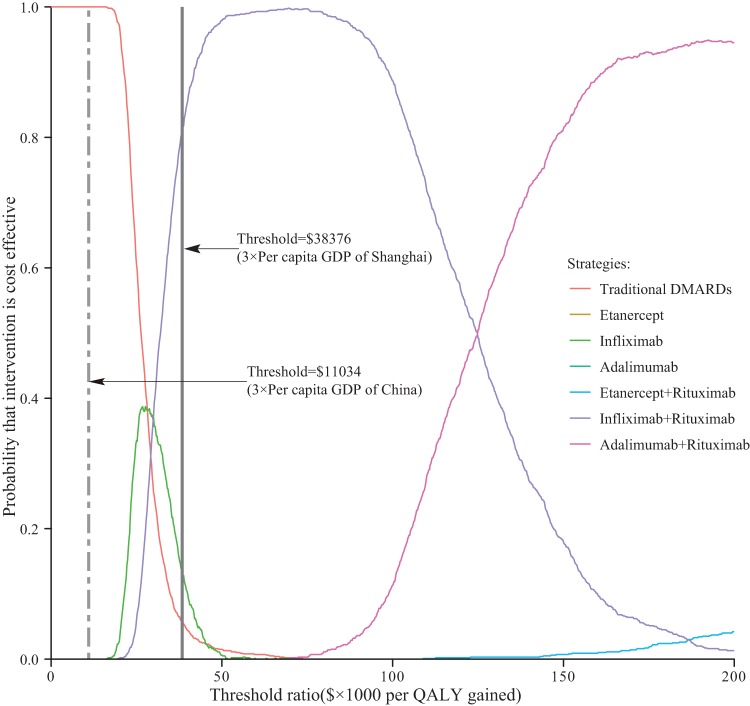
Cost-effectiveness acceptability curves of 7 competing strategies corresponding to probabilistic sensitivity analysisusing an ACR20 threshold. The bold vertical two-dash and solid lines represent the thresholds of 3×the per capita GDP of China and Shanghai per QALY gained, respectively.

Using the efficacy endpoint of ACR20, ACR50 and ACR70, we explored the three alternative scenarios for our probabilistic sensitivity analyses. When 3×the per capita GDP of China ($11,034) was used as a possible willingness to pay threshold, all simulations in the three scenarios fell to above of the line, which indicates that the ICERs yielded by biological therapies are greater than $11,034 per QALY (Figure 4). Cost-effectiveness acceptability curves also showed tDMARD strategy was the most cost-effective intervention when the threshold was equal to 3×the per capita GDP of China (Figure 5). Because each city of China has the power to set the coverage of drugs, those relatively developed cities, such as Shanghai and Beijing, would have the potential capacity to cover biological agents. The results of probabilistic sensitivity analyses showed that majority simulations (nearly 90%) in three scenarios for infliximab and infliximab + rituximab strategy were below the threshold of 3×the per capita GDP of Shanghai City ($38,376) per QALY (Figure 4). Acceptability curves indicate that the probability of cost-effectiveness produced by infliximab + rituximab strategy was nearly 81%, which was far higher than the other 6 strategies (Figure 5).

## Discussion

A systematic review has suggested that biologics are not cost effective in comparison with tDMARDs for RA at a cost-effectiveness threshold of $50,000 per QALY [9]. However, this threshold is potentially unsuitable for developing regions. The objective of this study was to evaluate the cost-effectiveness of biological therapies to measure the cost per QALY gained through the use of biological therapies compared with tDMARDs in the Chinese healthcare system. To our knowledge, this is the first analysis of comparing the cost-effectiveness of different treatment strategies for moderate to severe RA after the failure of at least two tDMARDs in a health resource-limited setting [9]. The mathematical model was used to simulate the potential clinical treatment sequences by incorporating data from varied sources.

In the current analysis, we identified the cost-effectiveness efficiency frontier in 7 potential treatment sequences: tDMARD → infliximab→infliximab + rituximab→adalimumab + rituximab→etanercept + rituximab. Infliximab is the most cost-effective option with the lowest cost and health benefit, and the etanercept + rituximab strategy offers the greatest health benefit with the most expensive cost. The notably lower withdraw probabilities in etanercept based strategies are the potential reason contributing to the much higher ICERs and QLAYs than infliximab based strategies. Based on the Weibull survival drug model, the median drug survival times of etanercept and infliximab strategy were 5.5 and 2.5 years, respectively. Because of the lower withdraw probability of etanercept than infliximab, more patients in etanercept strategy would remain in the state of receiving etanercept therapy during the same timeframe, which would lead to more QALYs and higher cost than infliximab. It is important to note that the ICERs of biological therapies over tDMARDs all meet the cost effectiveness threshold of $50,000 or $100,000 per QALY, which indicates that the TNF inhibitors are cost effective after tDMARD failure [3], [32]; however, these thresholds are not strict [33]. Our base case and probabilistic sensitivity analyses (PSA) result indicates that biological therapy may not be an attractive economic option for China because these ICERs are all higher than 3×the per capita GDP of China. For those patients living in relatively developed regions of China, infliximab initiated treatment followed by rituximab is a potential alternative option. For example, 3×the per capita GDP of Shanghai ($38,376) is higher than the ICER ($26,562.4) of infliximab + rituximab strategy over tDMARD. Nearly 90% RA patients receiving infliximab + rituximab strategy could achieve cost-effectiveness. Because our model showed that ICERs decrease when loss of work capacity was taken into account as an indirect cost, we could suggest that the ICER would become lower where the average wage was higher than the national average, such as in Shanghai City.

At present, Yisaipu®, a biosimilar versions of Enbrel® (etanercept), is already being marketed in China, which was nearly 3 times lower than the reference product.[34]. When the efficacy and safety profile of Yisaipu® was assumed to be same with Enbrel®, the cost and health outcomes would be $77,815.3 and 8.22 QALYs according to our model, respectively. The ICER of Yisaipu® against tDMARD strategy was $26,387.8, which was the even lower than the infliximab strategy. Although we noticed that the primary amino acid sequence of Yisaipu® was identical to the original product, the efficacy and safety of the biosimilar comparing with originally licensed biopharmaceutical is still need to be further investigated as mentioned by Gu N *et* *al* [35]. An updated analysis would be necessary when the data of non-inferior trial between the Yisaipu® and Enbrel® product is available.

To compare with a tDMARD sequence in patients who failed ≥2 tDMARDs, the published literature shows that treatment with TNF inhibitors resulted in median ICERs that were $75,000/QALY (range $72,000–$134,000/QALY) for etanercept + methotrexate, $133,000/QALY (range $80,000–$378,000/QALY) for infliximab + methotrexate, and $79,000/QALY (range $60,000–$175,000/QALY) for adalimumab + methotrexate [9]. Comparing our result with the above results is not straightforward because the design of the study, characteristics of the cohort, and health resource utilization setting are different. The ICERs in the published studies seem to be somewhat higher than ours, which seems mostly due to our QALY gain. The potential reasons for these differences might be partly due to the lower probabilities of withdrawal for tDMARDs (the withdrawal probability for leflunomide, gold and cyclosporine in our analysis were 20.7%, 10.6% and 25.3%, respectively, and in the study by Bansback *et* *al.* were all 27% [10]. Our results are consistent with those of Brennan *et* *al.* [17], who found that QALYs gained by etanercept and tDMARDs were7.53 5.87, respectively. The estimated drug costs for biotherapy in the current analysis were relatively higher than the previous studies. The most likely reason is the higher cost of etanercept, infliximab, and adalimumab in China, which when compared to Europe, is higher by nearly 80%, 45% and 45%, respectively [10]. Because the cost for biotherapy in total cost was over 90% in our result, it is expected that any factors which could affect the cost of biotherapeutic agents would have a substantial effect on the final results. Our one-way sensitivity analysis demonstrated that the discount rate and cost of infliximab were the second and fourth sensible parameter, respectively. There is a consensus in the literature that the likelihood of drug survival with infliximab is inferior to other TNF-α antagonists, so we examined the impact of long-term drug survival probability on the model outcome [36]. The results from the one-way sensitivity analysis showed the effect was limited (Figure 3).

Rituximab is the first-in-class agent for treatment of RA following the failure of TNF inhibitors [37]. Several published studies have indicated that rituximab in the treatment of RA was a cost-effective treatment alternative with ICERs from $18,348to $41,059 per QALY in comparison with the switch between TNF inhibitors as second line biological treatment [24], [25], [38]. The current analysis showed that adding rituximab to the treatment sequence could increase the health outcomes with higher ICERs. Compared to the etanercept and adalimumab strategies, the decrease inICERs of etanercept + rituximab and adalimumab + rituximab strategies were $10,934.8 and $6,900.7, respectively. For the infliximab + rituximab strategy, adding rituximab led to a modest increase in ICER of $2,218.2. These results suggest that adding rituximab followed by the inadequate response of TNF inhibitors in the Chinese setting might be an attractive cost-effective option. One-way sensitivity analysis has demonstrated that the cost of rituximab was the sixth most sensible parameter, decreasing the cost would yield more net health benefits. If the producer designates a lower price, we think rituximab would be more widely used for RA in clinical practice.

Several limitations exist in the current analysis. First, because there are no clinical trials that directly compare biotherapeutic agents for RA, the indirect method was employed to estimate relative efficacy data from separate studies in this analysis, possibly resulting in some bias. Results from one-way sensitivity analysis have shown that ACR20 achieved by infliximab was the fifth greatest effector. Direct comparative evidence could increase the certainty of our modeling results. However, the widespread accepted method has minimized the bias. If the data of heal-to-head trial is available, this economic analysis should be updated. Second, Chinese RA patients always discontinue the use biotherapy due to the high cost. The current analysis did not measure the effect of discontinuation on the final economic results because of the absence of data investigating the pattern of discontinuation and the effect on health outcomes. Taking into account that the main aim of our study was to the supply information to policy decision makers, this limitation could be neglected because discontinuation would disappear once the biotherapeutic agent becomes covered by healthcare systems. Third, due to a paucity of well-designed clinical trials for RA in China, the clinical data were mainly derived from abroad, which might produce bias on the final result. For example, the inpatient day of RA patients was estimated based on the HAQ score derived from a Swedish study. Based on the opinion of Chinese rheumatologists, the inpatient day used in current model was underestimated because most of Chinese RA patients would not be admitted to hospital due to limited health resources. Fortunately, the inpatient day has a relatively small impact on the robustness of the model. According to expert opinion, parameters which have substantial impact, such as the efficacy and withdrawal, were assumed to be similar with the trials conducted abroad. Finally, the current analysis did not represent all possible treatment sequences. According to the recommendation of guidelines [4], [39], switching to another TNF inhibitor or rituximab is the reasonable alternative. The current analysis excluded the option of switching to another TNF inhibitor because published studies have demonstrated that switching to rituximab was more cost-effective than switching to another TNF inhibitor; meanwhile, our analysis suggested that inserting rituximab into a TNF inhibitors strategy could decrease the ICER. For the above-mentioned reasons, and to simplify the model, we found it is necessary to analyze the economic outcome of switching to another TNF inhibitor after failure of one TNF inhibitor. However, because the results of this analysis reflected the general clinical practice of treatment for RA in China, we believe that this analysis can provide helpful information for Chinese health policy decision makers.

## Conclusions

In the Chinese healthcare setting, tDMARD was the cost-effective alternative option in the Chinese healthcare setting. However, in some relatively developed regions in China, infliximab and rituximab may be relatively cost-effective first-line RA treatments.
